# Imaging and Endovascular Treatment of Bleeding Pelvic Fractures: Review Article

**DOI:** 10.1007/s00270-018-2071-4

**Published:** 2018-09-17

**Authors:** Diederik J. Wijffels, Diederik O. Verbeek, Kornelis J. Ponsen, J. Carel Goslings, Otto M. van Delden

**Affiliations:** 1 Department of Radiology, Academic Medical Center (AMC), University of Amsterdam (UvA), Meibergdreef 9, 1100 DD Amsterdam, The Netherlands; 2 Trauma Unit, Academic Medical Center (AMC), University of Amsterdam (UvA), Amsterdam, The Netherlands; 3 Department of Surgery, Medisch Centrum Alkmaar (MCA), Alkmaar, The Netherlands

**Keywords:** Embolization, Trauma, Pelvic fracture, Imaging

## Abstract

Pelvic fractures are potentially life-threatening injuries with high mortality rates, mainly due to intractable pelvic arterial bleeding. However, concomitant injuries are frequent and may also be the cause of significant blood loss. As treatment varies depending on location and type of hemorrhage, timely imaging is of critical importance. Contrast-enhanced CT offers fast and detailed information on location and type of bleeding. Angiography with embolization for pelvic fracture hemorrhage, particularly when performed early, has shown high success rates as well as low complication rates and is currently accepted as the first method of bleeding control in pelvic fracture-related arterial hemorrhage. In the current review imaging workup, patient selection, technique, results and complications of pelvic embolization are described.

## Introduction

Major pelvic fractures occur in 4–9% of patients with blunt trauma, mainly high-speed traffic accidents or falls from height [1]. Considerable force is required to disrupt or fracture the pelvic ring, and over 50% of pelvic fracture patients incur multiple associated injuries [2, 3]. Major pelvic fractures are life-threatening injuries, with reported mortality rates up to 46% [1, 2, 4–6]. Mortality is caused by ongoing pelvic bleeding in as many as 42% of patients [3, 4]. However, bleeding may also occur from non-pelvic sources such as thoracic and abdominal injuries. To improve outcome, it is essential to rapidly identify the primary bleeding source. Plain films of chest and pelvis, along with focused assessment with sonography for trauma (FAST), have long been the mainstay of initial imaging assessment in hemodynamically unstable trauma patients, whereas contrast-enhanced CT (CECT) has generally been reserved for hemodynamically stable patients. CT has many advantages over FAST, and advances in CT technology as well as improved access have increased the role of CECT in hemodynamically unstable patients [7]. CECT provides detailed information about the location and type of hemorrhage as well as the extent of associated injuries and guides decisions on treatment. Controlling pelvic and other sources of hemorrhage through endovascular approaches has proven its effectiveness and is still improving, due to advances in patient selection, imaging equipment and embolization techniques [6, 8–12]. In this article, we review the recent literature on optimal imaging assessment and algorithms as well as the technique, results and complications of endovascular treatment of pelvic fracture-related hemorrhage.

## Imaging Assessment

**Fig. 1:**
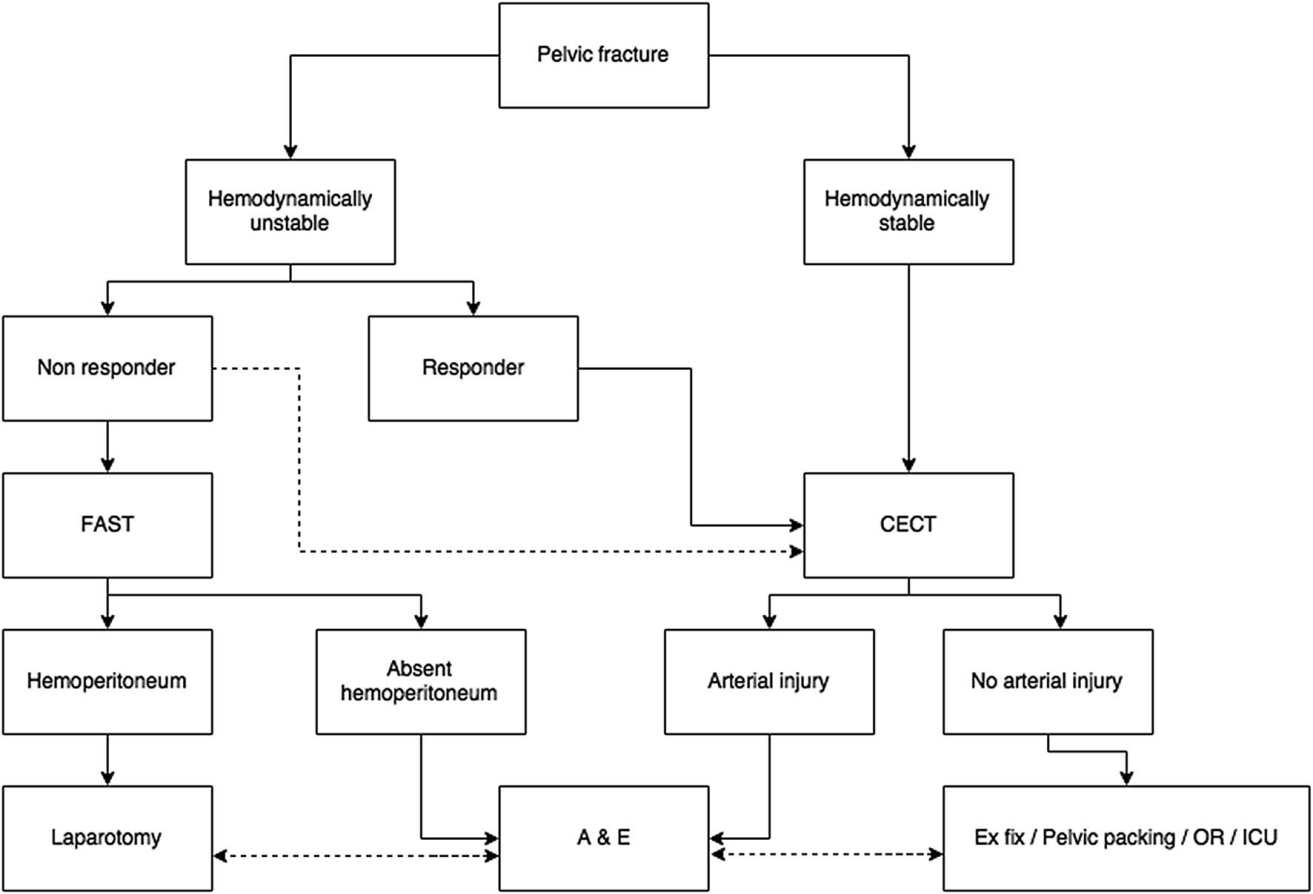
Local protocol for pelvic fracture workup. A&E: angiography and embolization. CECT: contrast-enhanced computed tomography. FAST: focused assessment with sonography in trauma. (Non-)responder: to fluid resuscitation. Dashed line CECT: if hemodynamics allow. Other dashed lines: if required due to clinical condition

Various guidelines have been described with treatment decisions largely driven by hemodynamic status and imaging findings [6, 13–16]. Our local algorithm is shown in Fig. 1. Imaging is crucial to differentiate abdominal from pelvic bleeding as well as venous from arterial bleeding as these factors determine the need for endovascular versus surgical intervention.

Over 90% of pelvic fractures are accurately diagnosed on plain films [17]. FAST detects hemoperitoneum with high sensitivity (90–93%), as a sign of significant organ injury [18]. In hemodynamically unstable pelvic trauma patients, FAST is especially useful to identify intraperitoneal bleeding which may require immediate laparotomy.

FAST is unable, however, to reliably detect retroperitoneal hematoma, the presence of ongoing bleeding, the exact location of hemorrhage, and to differentiate arterial from venous bleeding [18].

CECT is a much more accurate diagnostic test for the detection of arterial pelvic bleeding. The sensitivity of contrast extravasation (“blush”) indicating arterial leakage is 80–90%, specificity 85–98% and accuracy 87–98% [14, 19–22]. Furthermore, an extensive and detailed map is provided on location and extent of all injuries including all pelvic bony and vascular anatomy including relevant anatomic variants.

Currently, immediate whole-body CT has become the diagnostic modality of choice in many trauma centers, even in hemodynamically unstable patients. Compared to conventional workup with selective use of CT, it has been shown to save significant time [7, 24]. Retrospective studies and meta-analyses have shown an increase in the probability of survival of complex trauma patients with immediate whole-body CT scanning, although a recent multicenter randomized controlled trial failed to confirm this finding [23–26].

Various whole-body scanning protocols with sequential arterial and venous contrast phases and so-called split bolus protocols are used, in which one scanning phase combines features of arterial and venous phases. Scans are typically performed from the chest aperture to the pelvis with the arms raised over the head using 80–120 mls of contrast medium at injection rates of 4–6 mls./s. Image quality is reported to be better in sequential phase protocols compared to split bolus, but some studies suggest that venous phase imaging alone is reliable in detecting arterial hemorrhage, with sensitivity up to 100%, questioning the benefit of an extra-arterial phase, compared to single venous or split bolus [27, 28].

Other relevant imaging findings of CT include vascular injuries such as vasospasm, false aneurysm, arteriovenous fistula or completely dissected arteries, so-called vascular “cutoff’s.” A large amount (> 500 mL) of retroperitoneal hematoma on CT has a 45% probability for the presence of arterial injury and large transfusion requirement [29, 30].

## Angiography and embolization

### Indications

Pelvic fracture bleeding is most commonly of venous or bony origin [31, 32]. Less than 5% of patients have proven arterial injury, increasing to 59% in mechanically unstable pelvic ring fractures [1, 5, 6, 8, 10, 13, 33–35]. Furthermore, pelvic arterial bleeding is found in over 70% of pelvic fracture patients with transient or no response to fluid resuscitation (57). Angiography and embolization (AE) are currently accepted in many trauma centers as the preferred method for controlling pelvic arterial hemorrhage [8, 9, 14].

Indications for angiography include the presence of a (major) pelvic ring fracture with ongoing clinical signs of hemorrhage in the absence of a non-pelvic (thoracic or abdominal) bleeding source. Importantly, angiography should also be considered in patients with continued bleeding whose prior imaging did not demonstrate pelvic arterial bleeding [10–13, 33, 35].

Some authors advocated AE in the presence of a contrast blush irrespective of the patient’s hemodynamic status [3, 19–22] and in the presence of other vascular injuries such as a large pelvic hematoma, false aneurysms or “cutoff’s” may also require AE. Yet, the need for angiography in hemodynamically stable patients and a small contrast blush is a matter of continued debate. As a result of improvements in CT technology and the increased use of CT, more vascular injuries are found. Some authors have shown that not all contrast blushes seen on CT required embolization, and conversely, some patients without contrast extravasation required angiography [33, 36, 37]. Nevertheless, CT remains the gold standard for determining the presence of ongoing bleeding. In our institution, patients are generally directly referred for AE when a contrast blush is detected, with pelvic binders usually already placed. Depending on the hemodynamics and the fracture type and severity, AE is preceded or followed by external fixation of the pelvis given that AE has also been shown to be effective in patients failing to stabilize after external fixation (70).

Typically, injury is found to the internal iliac artery (IIA) or its branches [6, 14]. To some extent, pelvic fracture type may be related to the location of the vascular injury, with antero-posterior or so-called open book-type fractures being associated with bleeding from the posterior division of the IIA, and lateral compression or side impact fractures correlated with hemorrhage from the anterior division of the IIA. From the posterior branches of IIA, the superior gluteal artery and the lateral sacral artery are commonly injured. The superior gluteal artery is at risk with fractures involving the greater ischiadic notch [38]. Most commonly injured arteries from the anterior division of the IIA are the internal pudendal and the obturator artery [10, 14, 30]. High-grade vertical shear-type fractures are most often associated with arterial bleeding [6].

### Access

The presence of a sheet wrap or pelvic binder usually does not prevent gaining access to the common femoral artery for angiography. Alternatively, a brachial artery approach can be used. Ultrasound guidance is useful for obtaining vascular access, especially in hypotensive patients with collapsed vessels and no palpable femoral pulse. Placement of a 5-Fr sheath suffices in most cases.

### Angiographic technique

Procedures are generally performed with continuous monitoring of vital signs, and general anesthesia or sedation is not mandatory.

Direct selective catheterization of the IIA is mandatory as most arterial injuries are to the IIA or its branches [6, 14]. Both IIAs can usually be selected from a unilateral femoral access with standard 4 of 5-Fr diagnostic catheters. If the location of the hemorrhage is known, gaining contralateral access is advised to facilitate selecting the IIA. CT findings should act as a guide and should avoid unnecessary imaging and use of contrast, as trauma patients are prone for developing contrast-induced nephropathy (CIN) [39]. Obtaining an aortogram with a standard pigtail catheter is indicated only when the bleeding source is not detected during selective series of the IIA’s or when the patient does not stabilize after embolization of a bleeding source from the IIA’s. Lumbar arteries and branches of the external iliac artery should additionally be selected based on CT findings. Extravasation from the common femoral artery or side branches or contralateral vessels is uncommon but should be considered in the absence of other bleeding sources. When active extravasation is not detected at angiography, despite a contrast blush on CT, hemorrhage can be provoked by powerful selective hand injections of the suspected vessel, disrupting recently formed unstable clots. Normal blood pressure should be maintained in order to avoid obscuring the hemorrhage. However, normalization of blood pressure by aggressive resuscitation can also lead to rebleeding or disruption of newly formed blood clots elsewhere, so caution should be exercised. Bladder catheterization may be helpful to prevent obscuring the bleeding due to a contrast-filled bladder. Subtraction artifacts due to bowel movements may hamper visibility, and unsubtracted images can be helpful.

Besides direct contrast extravasation, other signs of arterial injury, such as false aneurysms or “cutoff’s,” should also be identified and treated as these have a high propensity for rebleeding when left untreated.

### Embolization technique

**Fig. 2:**
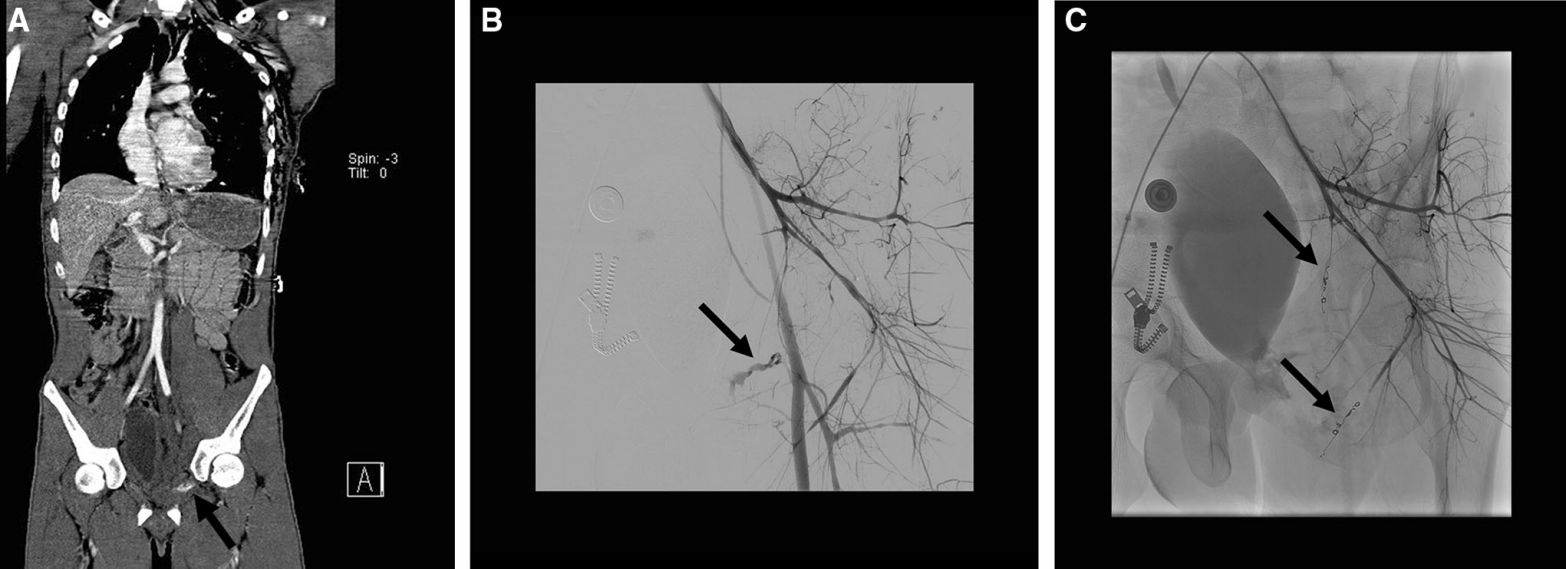
**A** coronal whole-body CECT of a hemodynamically stable trauma patient with a fracture of the left superior pubic ramus with pelvic hematoma and a contrast blush (black arrow). **B** Subsequent selective angiogram of the left internal iliac artery (with retrograde filling of the external iliac artery) shows constricted vessels and a focal contrast extravasation “blush” (black arrow) suitable for superselective embolization with coils and **C** selective angiogram of the left internal iliac artery after selective embolization with coils (black arrows)

**Fig. 3:**
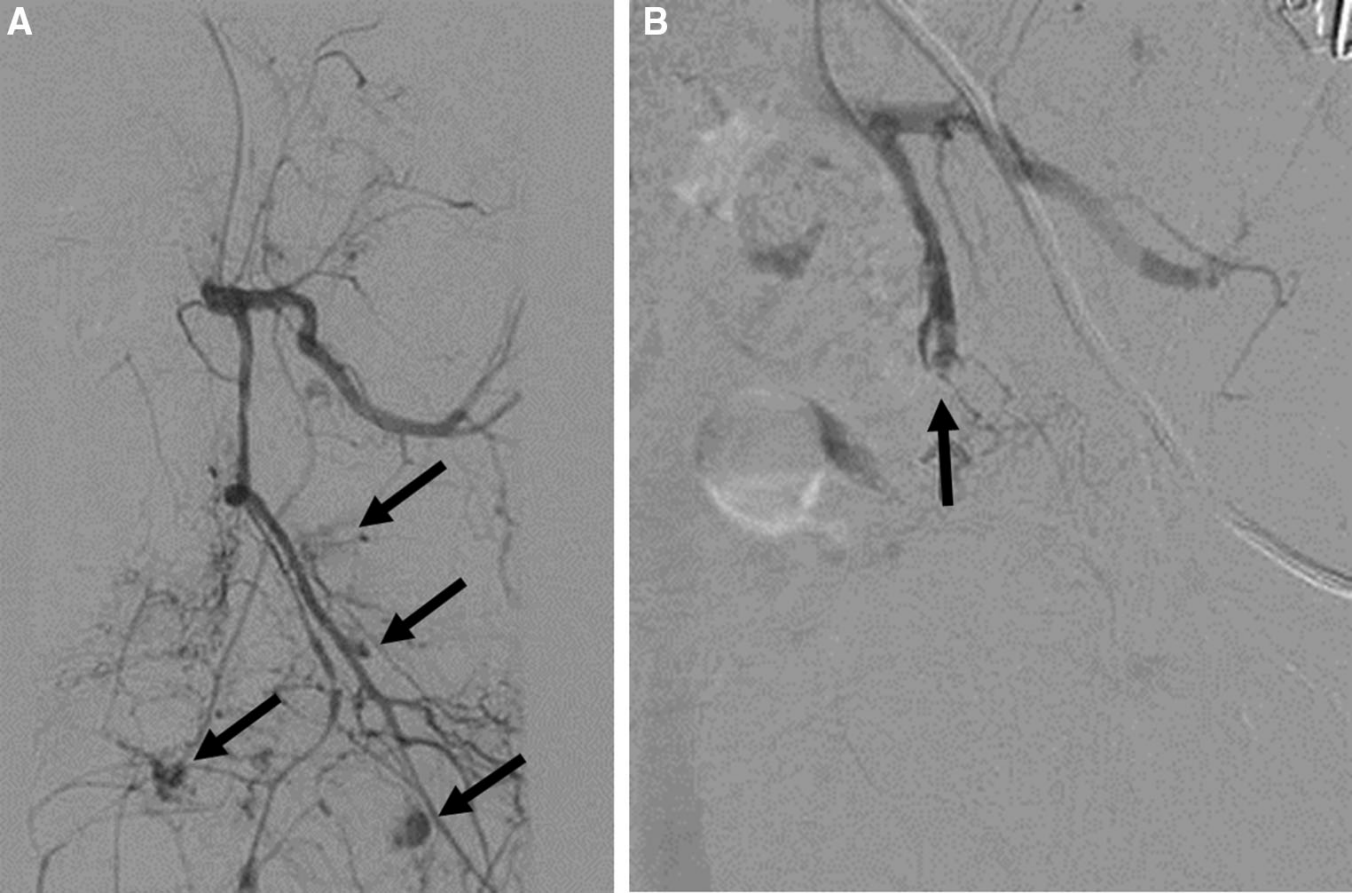
**A** Selective angiogram of the left internal iliac artery in a hemodynamically abnormal patient with pelvic fracture shows many areas of contrast extravasation (black arrows) from the anterior division not suitable or superselective embolization, and **B** selective angiogram of the left internal iliac artery shows complete occlusion (black arrow) of the anterior branch after embolization with gelfoam

Depending on the hemodynamic status of the patient and the time pressure, embolization is best performed selectively or non-selectively. Time permitting, selective embolization is preferred when there are only one or a few focal bleeding vessels (Fig. 2), when hemodynamics are stable. Superselective embolization, which often requires use of 2–3-Fr micro-catheters through a coaxial system, is technically more demanding and therefore time-consuming and is associated with a higher incidence of recurrent pelvic arterial hemorrhage [13]. Non-selective embolization (Fig. 3) is less time-consuming and can be performed using standard 4 or 5 Fr. catheters is preferred in hemodynamically unstable patients with ongoing hemorrhage. Non-selective unilateral embolization of the whole IIA or of the entire posterior or anterior division of the IIA can safely be performed in an emergency or if multiple vascular injuries or diffuse hemorrhage is found, which may occur in up to 40% of cases [10, 34]. Non-selective bilateral embolization of the entire IIA is a last resort for severe bleeding, but a widely accepted method without significant complications [40]. Superselective bilateral distal embolization is best avoided, as collateral blood supply from the contralateral IIA would be compromised, leading to an increased risk of tissue necrosis. Cessation of contrast extravasation from the affected vessel during the procedure is proof of technical success.

### Embolic agents

Both resorbable embolic agents gelatin sponge and non-resorbable embolic agents (stainless steel or platinum coils, vascular plugs and liquids agents) can be used for embolization. Typically, resorbable gelatin sponge slurry is injected to avoid permanent arterial occlusion and ischemia. Gelatin sponge slurry is an emulsion of gelatin sponge (cut to small cubes or torpedoes) and contrast agent, produced by mixing them. It can easily be injected through regular diagnostic 4- or 5-French catheters or a micro-catheter using a 1-ml syringe. Advantages are the speed of use and the relative independence from coagulation, making it a suitable agent in patients with coagulopathy. As it is a relatively non-selective embolic agent, it is particularly useful in case of multiple bleeding vessels or diffuse bleeding. An additional advantage over other embolic agents is the fact that it is bio-absorbable allowing for recanalization of occluded vessels after some time.

Coils are appropriate embolic agents when there are only one or a few focal bleeding vessels and for treating false aneurysms or “cutoff” vessels [12]. The main advantage of coils is that they allow for very precise positioning. Both regular pushable (0.035″) fibered coils and micro-coils can be used although vessel occlusion is usually obtained more quickly with 0.035″ coils compared with micro-coils. The use of detachable coils is usually not required. A disadvantage of coils is the fact that their efficacy depends on the patient’s coagulation, as clot needs to be formed in the coils before hemostasis is achieved. In the presence of coagulopathy, which frequently occurs in trauma patients, a combination of coil placement followed by injection of gelatin sponge can be very useful. Vascular plugs share many properties with coils and can also be used in some situations, where use of micro-catheters is not required. Other less frequently used agents are liquid agents like glue, which can be used fast, both selectively and non-selectively and in coagulopathic patients, but operator experience is required to prevent reflux and nontarget embolization.

In rare situations, stent grafts may rarely be used in the femoral or external iliac arteries, but this is beyond the scope of the current review.

### Results and outcome

It is difficult to assess outcome of AE as comparison between series is hampered by differences in patient population and because the outcome is often determined by other factors then pelvic hemorrhage alone, such as associated traumatic injuries. No randomized controlled trials have been conducted, and it is unlikely that these will be performed. Nevertheless, AE is found to be highly effective. Technical success is between 90 and 100% [8, 12, 16, 41, 42], while clinical success rates in terms of improved vital signs and decreased transfusion requirement are in the range of 84–100% [12, 34, 41]. Repeat angiography rates for ongoing or recurrent bleeding range between 0 and 23%, with a recent review reported 10% [13, 35, 41, 43]. Mortality rates among embolized patients vary widely between 4 and 56%, but a low number of reported deaths are due to exsanguinating pelvic hemorrhage (Table 1) [2].

**Table 1:** Overview of the recent literature analyzing pelvic fracture-related hemorrhage

References	P#	Angio	Embol.	T.S.	C.S.	Mortal.	Repeat	Compl	OM	PRM
Agolini [8]	806	35 (4.3%)	15 (43%)	15 (100%)	100%	7/15 (47%)	0	NA	74/806 (9.2%)	0
Wong [9]	507	22 (4.3%)	17 (77%)	17 (100%)	100%	3/17 (17.6%)	0	0	NA	0
Velmahos [12]	100	100 (100%)	80 (80%)	95%	NA	2 (14%)	4 (5%)	5 (6.3%)	NA	0
Cook [6]	150	23 (15%)	18 (78%)	18 (100%)	16 (89%)	10/18 (56%)	NA	2 (11%)	33/77 (43%)	0
Hagiwara [14]	234	81 (35%)	61 (75%)	NA	NA	13/61 (21%)	NA	NA	13/234 (5.6%)	NA
Kimbrell [11]	1017	92 (9%)	55 (60%)	55 (100%)	NA	10/55 (18%)	7/55 (13%)	4/55 (7.3%)	14/92 (15%)	NA
Shapiro [43]	678	31 (4.6%)	16 (52%)	*17/20 (85%)*	NA	2/16 (13%)	7/31 (23%)	NA	66/678 (9.7%)	NA
Fangio [34]	311	32 (10.3%)	25 (78%)	*24/25 (96%)*	21 (84%)	9/25 (36%)	0	1 (4%)	10/32 (31%)	NA
Totterman [10]	1260	46 (3.7%)	31 (67%)	29/31 (94%)	29/31 (94%)	5/31 (16%)	3/31 (9.7%)	0	NA	0
Verbeek [37]	217	58 (26.7%)	48 (83%)	46/48 (96%)	46/48 (96%)	2/48 (4.2%)	5/58 (8.6%)	NA	69/217 (32%)	2/48
Salim [55]	603	137 (23%)	85 (62%)	85/85 (100%)	NA	10/85 (11.8%)	NA	1 (1.2%)	54/603 (9%)	NA
Osborn [44]	20	20 (100%)	13 (65%)	NA	NA	NA	NA	NA	6/20 (30%)	2
Fang [13]	964	174 (18%)	140 (80%)	140/140 (100%)	NA	26/140 (18.6%)	26/140 (18.6%)	0	NA	12
Jeske [15]	1476	42 (2.8%)	41 (98%)	41/42 (98%)	NA	13/41 (31.7%)	3/41 (7.3%)	10 (24%)	NA	0
Hauschild [3]	152	17 (11.2%)	17 (100%)	17/17 (100%)	17 (100%)	3/17 (17.6%)	0	6 (35%)	NA	0
Tanizaki [46]	140	68 (49%)	68 (100%)	NA	NA	12/68 (17.6%)	0	0	NA	1
Ierardi [41]	168	NA	160	160 (100%)	133 (95%)	15 (9.4%)	3 (1.9%)	0	NA	7
Lustenberger [16]	173	16 (9.2%)	16 (100%)	16 (100%)	16 (100%)	4/16 (25%)	NA	0	NA	0

*NA* data not available, *P#* number of pelvic fractures, *Angio* angiography performed, *Embol.* embolization, *T.S.* technical success, *C.S.* clinical success, *Mortal.* mortality in embolization group, *Repeat* angiography, *Compl.* number of complications, *OM* overall mortality, *PRM* pelvic-related mortality

External fixation of the pelvis to treat bleeding effectively reduces fracture displacement and decreases the volume of the pelvis, but this has not been shown to result in arterial hemorrhage control and pelvic packing compared with AE showed no significant differences in mortality and blood transfusion [44]. Others report favorable results combining both methods or using pelvic packing alone [15, 45].

An important predictor for the outcome in pelvic trauma patients is the time between arrival in the trauma bay and angiography suite. Tanizaki showed that patients who arrived late in angiography suite had a higher mortality than those arriving within 60 min [46]. Agolini found a mortality of 14% if the patient’s arrival in the angiosuite was within 3 h, increasing to 75% after 3 h [8]. In patients undergoing repeat angiography, blood transfusion rate, ICU stay and mortality were significantly higher than in those who underwent a single procedure [10, 12, 13, 35, 43]. Increased age, initial hemodynamic instability and the need for blood transfusion have also been also positively correlated with an increased mortality [8, 9, 14].

### Complications

In more than 95% of the embolized patients, no significant complications directly related to the procedure are observed [12, 33, 34, 42, 47, 48].

Angiography complications including contrast allergy, contrast-induced nephropathy (CIN) and puncture site-related complications (hematoma, false aneurysm and infection) have been reported, but data are very limited and the actual prevalence may be higher due to underreporting [6, 12].

CIN has been reported in 5.1% of trauma patients after CECT [49], but recent studies show an incidence of 20–24% in trauma patients undergoing CECT and subsequent AE [15, 39]. Hypovolemic shock, multi-organ failure and volume depletion may also contribute to renal impairment.

Embolization complications are predominantly ischemic. Ischemia and necrosis of gluteal muscle, sacral skin and bladder wall, uterus, femoral head and leg have been reported [6, 15, 34, 50, 51]. Gluteal muscle ischemia or necrosis has a reported incidence of 3–6% [6, 15, 47, 48, 52], but the initial trauma itself also disrupts local vasculature. Although embolization of the IIA may worsen recovery, gluteal ischemia has only been reported after bilateral embolization of the IIA. Erectile dysfunction has frequently been described after bilateral embolization [47], but is probably due to the injury itself rather than the embolization [48, 53, 54].

Neurologic complications including lower leg paresthesia and paresis have been described, but no significant differences have been observed between embolized and non-embolized patients [3, 48].

## Conclusion

Pelvic angiography and subsequent embolization is a safe, rapid and effective technique for patients with pelvic fracture-related arterial hemorrhage in both hemodynamically stable and unstable patients. The choice between selective or non-selective embolization is dictated by the level of urgency and angiographic findings, and a variety of embolic agents can be used. CECT is crucial to show and localize arterial bleeding.
